# Deep Learning Image Processing Models in Dermatopathology

**DOI:** 10.3390/diagnostics15192517

**Published:** 2025-10-04

**Authors:** Apoorva Mehta, Mateen Motavaf, Danyal Raza, Neil Jairath, Akshay Pulavarty, Ziyang Xu, Michael A. Occidental, Alejandro A. Gru, Alexandra Flamm

**Affiliations:** 1 Vagelos College of Physicians and Surgeons, Columbia University, New York, NY 10032, USA; ahm2178@cumc.columbia.edu; 2 School of Medicine, Duke University, Durham, NC 27710, USA; 3 The Ronald O. Perelman Department of Dermatology, New York University Grossman School of Medicine, New York, NY 10016, USAmichael.occidental@nyulangone.org (M.A.O.); alexandra.flamm@nyulangone.org (A.F.); 4 Department of Dermatology Columbia, University Irving Medical Center, 622 W 168th St, New York, NY 10032, USA

**Keywords:** dermatopathology, deep learning, convolutional neural networks (CNNs), vision transformers (ViTs), foundation models, whole-slide imaging (WSI), dataset bias, good machine learning practice (GMLP)

## Abstract

Dermatopathology has rapidly advanced due to the implementation of deep learning models and artificial intelligence (AI). From convolutional neural networks (CNNs) to transformer-based foundation models, these systems are now capable of accurate whole-slide analysis and multimodal integration. This review synthesizes the most recent advents of deep-learning architecture and synthesizes its evolution from first-generation CNNs to hybrid CNN-transformer systems to large-scale foundational models such as Paige’s PanDerm AI and Virchow. Herein, we examine performance benchmarks from real-world deployments of major dermatopathology deep learning models (DermAI, PathAssist Derm), as well as emerging next-generation models still under research and development. We assess barriers to clinical workflow adoption such as dataset bias, AI interpretability, and government regulation. Further, we discuss potential future research directions and emphasize the need for diverse, prospectively curated datasets, explainability frameworks for trust in AI, and rigorous compliance to Good Machine-Learning-Practice (GMLP) to achieve safe and scalable deep learning dermatopathology models that can fully integrate into clinical workflows.

## 1. Introduction

Dermatopathology faces a dual pressure: an increasing incidence of skin diseases globally and a rising shortage of board-certified dermatopathologists internationally [1,2]. While technological innovations such as the digital slide scanner and whole-slide imaging may aid in alleviating these bottlenecks, the true inflection point in scaling dermatopathology may exist in deep learning models. The introduction of deep learning models within dermatology was catalyzed by Esteva et al.’s landmark 2017 CNN which achieved dermatologist-level dermascopic performance [3]. Researchers translated this technology to histopathology. Early CNNs excelled at local feature extraction, but many models struggled with high granularity, gigapixel scales, and complex spatial context of whole-slide imaging. Since the advent of the first CNN in dermatopathology, the field has made significant advancements in three waves. First, we had the CNN dominated era: patch-based ResNet and Inception model derivates were able to reach non-inferior accuracy in binary melanoma tasks [4,5,6,7]. Next came the emergence of transformer models. Vision Transformers (ViTs) and attention mechanisms became capable of global context modeling on whole-slides, demonstrating superior performance in select tasks such as margin assessment and certain multi-class classification problems [8]. Most recently, the emergence of hybrid and foundation models such as graph transformers, CNN-ViT hybrids, and multi-billion parameter models trained on millions of whole-slide images (WSIs) may ultimately achieve multi-modal fusion and generalizability [9].

While algorithmic advances have dominated mindshare in deep learning dermatopathology, commercial platforms and products have implemented these techniques in real-world clinical settings. Products such as Proscia’s DermAI, PathAI’s PathAssist Derm, Aisencia’s Cutaneous80, and Paige’s PanDerm/PanCancer Detect are integrating AI triaging and clinical decision support with tight regulations such as involving a human-in-the-loop for each workflow. This means that prior to any official diagnosis communication with the patient, a board-certified dermatopathologist will review the AI analysis.

Herein, this review critically evaluates the technological advancements of deep learning in dermatopathology, dissects performance benchmarks of existing state-of-the-art models, and discusses key translational gaps. We also highlight the importance of diversity, explainability, and regulatory compliance that must be addressed before achieving autonomous and safe AI-augmented dermatopathology. While prior reviews have discussed aspects of deep learning in dermatopathology, our work differs by integrating the architectural evolution into a single historical narrative—comparing CNN, transformer, hybrid, and foundation models—while also highlighting clinically deployed platforms and analyzing translational barriers such as workflow integration and regulatory approval. This dual emphasis on both technical evolution and clinical adoption provides a novel, pragmatic perspective for dermatologists and dermatopathologists considering real-world AI implementation.

### Methods: Narrative Review Approach

This review was conducted as a narrative review given the relative novelty of deep learning models in dermatopathology and the limited availability of peer-reviewed literature. Relevant articles were identified through PubMed and Google Scholar searches between 2017 and 2025, supplemented by searches of preprint servers (arXiv, bioRxiv) due to the rapidly evolving nature of the field. Keywords included “deep learning,” “dermatopathology,” “convolutional neural network,” “transformer,” “hybrid model,” and “foundation model.” Articles were included if they described the development, validation, or clinical deployment of deep learning models applied to dermatopathology or histopathology. Exclusion criteria were non-imaging AI applications or non-pathology image tasks. Because of the heterogeneity of study designs, results were synthesized descriptively rather than pooled quantitatively. The reliance on preprints is acknowledged as a limitation, but these works provide valuable insight into the state of the field in advance of peer-reviewed publication.

## 2. Historical Overview of Deep Learning in Dermatopathology

### 2.1. Convolutional Neural Networks (CNNs) in Dermatopathology

Convolutional Neural Networks (CNNs) have historically served as the foundational architecture of dermatopathology image analysis. A CNN consists of layers of convolutional filters that extract features and patterns (edges, textures, shapes) from images and assemble them into complex cellular or tissue structures. This hierarchical feature learning method allows CNNs to utilize raw pixel data to capture diagnostically relevant patterns [3,7]. In digital pathology workflows, pathology slide images are divided into small tiles (e.g., 256 × 256 pixels), each processed by a CNN, and the patch predictions are later aggregated to inform an overall diagnosis [10]. The approach of patches was instrumental in the first use cases of CNNs in dermatopathology. Datasets such as The Cancer Genome Atlas (TCGA) have supplied thousands of whole-slide H&E-stained images of melanoma and other various skin pathologies used to build robust CNN models [10,11]. Metrics such as sensitivity, specificity, accuracy, and area under the receiver operating characteristic (ROC) curve, in addition to inter-observer agreement amongst expert dermatopathologists, validate the performance of these models. Studies and novel models also use statistical tests to demonstrate non-inferiority to human specialists.

The first landmark application of deep learning came from Esteva et al. in 2017 demonstrated the potential of deep convolutional neural networks (CNNs) when they developed a model that achieved non-inferior diagnostic accuracy to dermatologists in the classification of various dermatologic malignancies, including melanoma, basal cell carcinoma, and squamous cell carcinoma [3]. This catalyzed the transition of CNNs into histopathology. Hekler et al. subsequently highlighted head-to-head performance statistics between a deep CNN and board-certified dermatopathologists on histopathologic melanoma versus nevus images. Compared to the 59% accuracy rate of 11 dermatopathologists (*p* = 0.016), the CNN outperformed significantly by reaching a 68% accuracy rate [4,5]. Xie et al. developed a CNN that achieved an AUROC of 0.986 with 93.8% sensitivity and 95.7% specificity in detecting melanoma on whole-slide histopathology images [12]. Their model either matched or exceeded the performance of 20 pathologists. Similarly, Wang et al. demonstrated a model for eyelid melanocytes tumor detection that achieved 100% sensitivity, 96.5% specificity, and 98.2% accuracy for whole-slide images compared to a mean accuracy of 92% (±6.2%) for a group of dermatopathologists [13]. Other models, such as CNN-random forest hybrids, which is a model that uses randomization to create large numbers of decision trees as inputs that allows for discovery of interactions between predictors, likewise achieved non-inferior sensitivity and specificity compared to human specialists [14,15]. Collectively, these studies established CNNs as a credible diagnostic tool for routine classification and clinical decision tasks in dermatopathology [16].

Despite their successes, CNNs exhibit important limitations. They are localized to their receptive field and excel at capturing fine details, but lack the ability to integrate global tissue architecture across gigapixel-scale slides [4,17]. This narrow focus increases the risk of overfitting to local signals and can limit generalizability to more complex or subtle diagnostic tasks. These shortcomings motivated the adoption of architectures such as transformers, which can model long-range dependencies and global context in whole-slide imaging [18].

### 2.2. Vision Transformers and Self-Attention Mechanisms

Vision Transformers (ViTs) represent a new generation of deep learning artificial intelligence models. Unlike CNNs, which process pixels in narrow, localized windows, ViTs operate by splitting an image into patches [19]. These patches are then processed in sequence, analogous to words in a sentence, using a process called self-attention. The self-attention mechanism allows the model to weigh relationships between any two random patches and capture long-range dependencies and the global structure of the slide [10,18,19].

Each patch of an image is embedded into a feature vector (the values of all features of a data point) and combined with positional encodings that retain spatial information. These vectors are then sent into multiple layers of attention heads that highlight informative regions in the context of the entire slide [10]. This process allows ViTs to build a global field of view that detects patterns that may span multiple parts of a slide: for example, recognizing that a minimal amount of atypical cells across a large area may indicate an early neoplastic lesions. Within two years of the introduction of ViTs to medical imaging, they frequently began outperforming state-of-the-art CNNs when trained on sufficient and appropriate datasets [8].

In one squamous cell carcinoma margin assessment case study, a ViT achieved an accuracy of 92.8% with an AUC of 0.93—significantly outperforming the leading CNN (InceptionV3), which reached an 86% accuracy on the same task at optimal performance [20]. These results highlight how robust ViTs are in analyzing pathology slides and their ability to surpass even the best, most thoroughly developed CNNs. Furthermore, in general skin lesion classification tasks, such as distinguishing between benign nevi, dysplastic nevi, melanoma, and seborrheic keratoses, ViTs exceeded CNN accuracy in several reports [19,20,21]. These studies highlight that ViTs are not merely incremental improvements, but a paradigm shift toward architectures capable of capturing tissue-wide context absent in CNNs. However, despite their strengths, they require vast datasets and do not generalize well. To address the limitations posed by data, investigators have begun identifying hybrid CNN transformer models for dermatopathology tasks [21].

### 2.3. Hybrid CNN-Transformer Models for Whole-Slide Analysis

Whole-slide histopathology images are typically on a gigapixel scale. As a result, hybrid models are the new focus due to the evident need for models to capture both fine-grained details and global tissue architecture to assess complex pathologies [10]. These approaches integrate CNN-style feature extraction techniques with transformer-based global contextual modeling. A common approach to deploying hybrid models is to use a CNN to convert each image patch into a feature vector and then use a transformer or attention network to aggregate information across all patches from a slide. In this way, the model can extract fine details while maintaining the global context of the slide. This approach leverages CNN’s low-level pattern recognition and the transformer’s ability to model relationships between various slide regions [10].

One example of a hybrid model for dermatopathology is the Graph Transformer (GTP), developed by Zheng et al. In this model, each WSI is represented as a graph of patch features (nodes with CNN-derived embeddings) fed into a transformer to predict the slide-level diagnosis. When tested on a dataset of 4818 WSIs for lung cancer, including TCGA, the model’s hybrid graph/ViT architecture achieved high three-class accuracy with 91% cross-validation accuracy. It justified its diagnostic predictions by highlighting histologic regions in the slide [10]. Nie et al. demonstrated another hybrid model that classifies patches and uses an attention mechanism to decide the slide’s class. This model achieved superior performance compared to senior pathologists in differentiating between benign, atypical, and malignant skin lesions with higher F1-scores on both internal and external test datasets [22].

These results ultimately emphasize the importance of combining local feature analysis from CNNs with global context from ViTs—emulating techniques used by human pathologists, such as scanning a slide at lower power and zooming in on regions of interest [23,24].

Advancements and research in hybrid architecture suggest that these models augment the abilities of human specialists in handling complex dermatopathology images. Using CNN models to encode morphology and ViT models to provide contextual cues, hybrid models may be used in high-acuity cases, such as tumor subtyping and margin assessment. For instance, HistoGPT is a hybrid transformer model trained on over 15,000 whole slide images that demonstrated very strong performance on dermatopathology classification tasks. Benchmarking results demonstrated that HistoGPT generated reports that matched the quality of dermatopathologists’ for common and homogenous malignancies when evaluated by natural language processing metrics and domain expert analysis [25]. A review of models can be seen below in Table 1, Table 2 and Table 3.

Taken together, CNNs established deep learning as a credible diagnostic tool, VITs expanded performance by capturing the global context, and hybrid/foundation models now integrate both local and global features. This stepwise progression underscores how architectural innovations have mirrored the needs of dermatopathology, from binary lesion classification to multi-class, multi-modal analysis.

## 3. Clinically Deployed Models in Dermatopathology

Artificial intelligence has emerged as a powerful assistive tool in diagnostic pathology. Deep learning models have demonstrated efficacy in detecting, analyzing, and classifying skin lesions, leading to increased performance and efficiency for providers and better diagnostic accuracy for patients [27]. With over 25 million skin biopsies analyzed every year in the United States alone, the increased reliability and accuracy of these models has become more readily accepted by both physicians and patients alike [28]. In this section, we will focus particularly on three leading examples of AI models that have demonstrated efficacy and are currently in clinical deployment, namely DermAI (Proscia Inc. Philadelphia, PA, USA), PathAssist Derm (Path AI), Aisencia’s Cutaneous80, and Paige PanCancer/PanDerm Detect. We will also focus on other promising AI models that are still under development, summarizing their key applications, performance, impact, and regulatory status.

### 3.1. DermAI (Proscia Inc.)

The first clinically deployed model to discuss is Proscia Inc.’s DermAI, which functions as an AI module that is directly integrated into the broader Proscia Concentriq digital pathology platform. This particular module aims to screen skin biopsies before they are passed to a licensed pathologist for professional review [29,30,31,32,33]. The DermAI module uses deep learning to automatically separate images of whole slide skin biopsies into broader diagnostic categories (such as basaloid, squamous, melanocytic, etc.) in order to triage high priority cases as well as cases that need additional provider review [34]. Since DermAI runs in the background for any incoming scans and assigns a confidence score, cases are more quickly sorted and rapidly distributed to pathologists with specialized expertise. Higher priority cases, like those involving potential malignancy, are flagged for immediate review. To further ensure accuracy, DermAI provides an independent AI assessment on every case, acting as a second read to catch any errors or discrepancies [34]. Overall, this has potential to reduce diagnostic delays and prevent costly errors, most significantly aiding those labs with the highest volumes and limited staff.

Proscia reports the testing process involved 20,000 dermatopathology slides that were used through a large multicenter study over both academic and commercial laboratories [29,30]. Exact performance metrics are not disclosed, but it is worthwhile noting many board-certified dermatopathologists, such as Dr. Kiran Motaparthi who served as director of dermatopathology at University of Florida, note that “DermAI demonstrates a high level of accuracy in categorizing common diagnoses,” which highlight the tool’s reliability and accuracy [29]. DermAI’s consistency in predictions across multiple real-world cases suggests that the performance is potentially approaching expert levels in its preliminary classification abilities [30]. The main strength of this tool surrounds its high sensitivity, as the model is trained to flag any suspicious lesions for additional review.

The DermAI model is integrated with the Proscia Concentriq AP-Dx platform, which in February 2024 received 510(k) clearance from the FDA [35]. Through this major milestone in regulatory processing, the Concentriq AP-Dx platform is now cleared for primary diagnosis in U.S. clinical settings when used in conjunction with the Hamamatsu NanoZoomer S360MD Slide scanner. This clearance comes after a multi-site (PathGroup, South Bend Medical Foundation, and Spectrum Healthcare Partners) clinical study that Proscia claims on their website demonstrated digital reads on the Concentriq AP-Dx were comparable to traditional glass side diagnosis in terms of accuracy. Additionally, they reported a major discordance rate of only 0.1% relative to ground truth [35]. The regulatory clearance of the core Concentriq system represents a paradigm shift in the clinical viability and trustworthiness of AI-augmented digital pathology.

Proscia’s DermAI and Concentriq AP-Dx is currently used by over 10,000 pathologists and scientists at over 14 of the top 20 pharmaceutical companies worldwide, and they additionally received regulatory approvals including CE-IVDR certification in the EU, licensure in Canada, and clearance in the United Kingdom [35]. Together, these tools have the potential to reduce turnaround times, optimize pathologist workload, and improve patient care, all which serve to validate the clinical and operational promise of digital AI-augmented dermatopathology.

### 3.2. PathAssist Derm (PathAI)

Another notable deep learning tool currently used in dermatopathology is PathAssist Derm, created by PathAI. This tool is currently deployed on PathAI’s AISight platform, which allows it to directly integrate with digital pathology systems to support decision-making processes for skin biopsies [36,37]. PathAssist Derm stands out in its ability to automatically rotate and register each fed image into a standard view, which allows histology slides to be scanned at any angle [36,38]. This eliminates a mundane task for pathologists or assistants who may prepare slides, as PathAssist Derm will automatically ensure a consistent presentation of the histology. This tool has been trained on a remarkably large dataset of millions of images from hundreds of thousands of patients, allowing it to be able to identify and categorize a wide range of skin lesion types [39]. The model is able to predict and categorize up to 17 different potential diagnoses, including more common malignancies (such as BCC and SCC), premalignant lesions such as actinic keratosis, benign mimickers such as lichenoid keratosis, and even melanoma. Due to its extensive ability to detect patterns in prepared histology slides, PathAssist Derm can highlight specific regions of interest that the pathologist can then analyze, as well as provide quantitative measurements (such as dimensions of a lesion or depth of invasion), which can be crucial for malignancy staging [36]. These features highlight the ability of PathAssist Derm in AI-driven morphometry, allowing pathologists and dermatologists to gather objective data from a slide that can then be verified and incorporated into the diagnosis.

Since PathAssist Derm was recently launched in February 2025, detailed performance metrics are not extensively published, although the system’s accuracy has claimed to have been internally validated extensively, with PathAI reporting “strong diagnostic accuracy” in multi-condition validation [39]. The model was trained with a high emphasis on identifying skin cancers, as it is of high clinical priority to not overlook any potentially malignant melanomas or carcinomas. In other words, PathAssist Derm’s algorithms similarly favor sensitivity, flagging any region that may be suspicious for cancer even at the potential cost for some false positives in an effort to ensure that high urgency cases are not missed [36]. Although this tool itself does not currently have significant publicly available data, related dermatology AI models have shown relative performance to dermatopathologists on much smaller datasets. With such a large training dataset, it would be expected that PathAssisst Derm would demonstrate similar or better performance with more robust generalizability, breadth, and reliability.

This tool is currently only deployed as a research and clinical decision support tool and is not FDA-approved for primary diagnosis. It is being offered to pathology laboratories and researchers under strict guidance that a human must review all AI findings to make the final diagnosis [39]. PathAI is currently in the process of clinical implementations and trials, and the company has stated that it is working towards FDA clearance. This path to approval will require the company to prove the safety and efficacy of the device, demonstrating that the model’s use improves outcomes without compromising safety. Based on current press releases from the company, PathAI’s approach contrasts with fully autonomous AI diagnostics, as PathAssist Derm is positioned as a support tool rather than replacing physician judgment, which could streamline regulatory acceptance as a Class II instead of Class III medical device [40]. The company has previously attained approval for other devices, so the expectation is that PathAssist Derm will also undergo rigorous validation and eventual approval.

This model could extensively benefit dermatopathology practice through more efficient slide review. Through automation of time-consuming tasks like slide orientation, magnification, and pre-screening for relevant features or landmarks, the manual workload for pathologists and laboratory staff is significantly reduced, saving valuable time and helping to manage heavy case-loads. The high sensitivity of the model can prevent critical cases from being buried amidst the noise, draw attention to areas of interest, and reduce total slide viewing time per case; the use of this tool can lead to faster diagnoses for patients with skin cancer and therefore improve outcomes [41]. This increased efficiency does not come at the expense of accuracy, as the AI should serve to act as a safety layer and help catch subtle findings that a human reviewer may overlook. With complex pathology involving a level of subjective decision-making, deep learning AI models such as PathAssist Derm can provide a level of standardized objective truth which can minimize variability between different pathologists. The additional quantitative outputs of the model can enhance pathology reports and downstream clinical decisions, and outside of direct diagnostics, could be used as a teaching and reference tool. Though still in the evaluation phase, the real-world impact of PathAssist Derm is poised to grow as more labs adopt digital pathology and integrate AI into routine practice.

### 3.3. Aisencia AI

Aisencia GmbH, a digital pathology company based in Germany, developed Cutaneous80, their AI-powered tool to detect and classify over 40 common and rare skin conditions on H&E-stained slides, including basal cell carcinoma, squamous cell carcinoma, melanoma, and Merkel cell carcinoma. The system was designed to address routine dermatopathology workflows with reported coverage of nearly 80% of daily diagnostic case volume [42]. It uses a patch-based multi-step modular pipeline optimized for large-scale triage in routine pathology practice. The model begins by dividing WSIs into tiles of 128 by 128 pixels and filters out non-tissue areas. Once quality control and color normalization are completed, it is passed through a ResNet-50 convolutional neural network to extract dense feature embeddings. The system then proceeds to route the extracted features through a suite of over 50 task-specific CNNs, which are trained to detect particular lesion types. Finally, tile-level outputs are aggregated using a multiple-instance learning (MIL) framework to diagnose the specimen [43,44].

A key differentiator of Aisencia’s approach is its strong emphasis on real-world workflow integration. The system includes features such as automated region of interest highlighting, smart case prioritization, and natural-language structured macro-report generation. These functions integrate with common digital pathology platforms, including PathPresenter, CoPath, NovoPath, and Epic Beaker, enabling rapid implementation in clinical laboratories [45]. The model flags potentially malignant lesions for review and pre-sorts cases into categories for subspecialty routing, offering substantial time savings and reducing manual triage burden.

Aisencia has also developed a specialized melanoma metastasis detection tool, designed for sentinel lymph node (SLN) histology, with high sensitivity for deposits greater than 0.1 mm. This model attempts to address a known diagnostic challenge—distinguishing micrometastases from benign nodal nevi—which remains a high-variability task even among experienced pathologists [46].

Aisencia’s AI tools have been validated in operational pilots across German and other European dermatopathology labs. Peer-reviewed performance metrics remain limited, but internal data and customer feedback suggests strong diagnostic concordance and high interobserver agreement. At the American Academy of Dermatology (AAD) 2025 conference, Aisencia’s abilities were showcased in a live production environment within PathPresenter and is actively being deployed within dermatopathology clinics internationally [47]. With continued expansion of lesion coverage and the development of modules for inflammatory dermatoses and tumor segmentation, Aisencia represents a pragmatic, workflow-conscious model of AI deployment that aligns well with the current regulatory and clinical realities of European dermatopathology practice.

### 3.4. Virchow/Paige PanDerm AI

The last major model is Paige’s PanCancer/PanDerm Detect model built by Paige AI on their initial Virchow model. The initial Virchow model was one of the largest and most comprehensive foundational models for computational pathology, which was developed jointly by Paige and Microsoft Research. It uses a vision transformer architecture with 632 million parameters and was trained on 1.5 million whole-slide images spanning 17 rare and common malignancies [9]. The model employs the DINOv2 base method and was fine-tuned for clinical tasks after pre-training. Evaluation of the initial model demonstrated specimen-level AUC of 0.949 for multi-cancer detection and an AUC of 0.937 when explicitly focused on rare cancers such as sarcomas and melanomas. This performance extended to both classification and biomarker prediction tasks [9].

Paige’s PanCancer/PanDerm Detect platform significantly expanded upon the Virchow model with Virchow V2, training on over 3 million digitized whole-slide images (WSIs) from more than 40 unique tissue types with 1.8 billion parameters [48]. The model represents one of the largest multi-organ histopathology datasets assembled for deep learning training. Due to the training scale, the model works on a tissue-agnostic architecture basis, which allows it to generalize across any organ without retraining on task-specific datasets [48,49]. Unlike traditional CNNs that use local convolution and patch-level prediction, PanCancer employs a self-attention methodology that allows it to build and perceive complex spatial relationships across the entire digitalized WSI. Self-attention allows the transformer model to identify subtle morphological patterns, capture long-range dependences, and recognize rare or atypical clinical presentations or edge cases that may elude more narrow models or even board-certified dermatopathologists [48].

One innovative approach the PanDerm model employs is multi-instance learning (MIL) for WSI interpretation. MIL treats a WSI as a collection of unlabeled image patches called instances, which are nested within a more prominent slide-level label called the “bag” [50]. Using MIL, the model can improve and learn from weakly labeled data. This is a fundamental skill and critical in histopathology as patch-level annotations are inaccessible due to the labor-intensive process of manually labeling slides. The MIL approach enhances interpretability by directing the model’s attention to diagnostically relevant regions in the WSI while minimizing noise from irrelevant tissue. Furthermore, PanDerm AI uses CNN feature extraction and transformer attention aggregation elements, rendering it an accurate hybrid model [48,50].

Yan et al.’s study highlights PanDerm’s effectiveness through evaluation with 28 datasets used in training past models (Table 4) [51]. They tested PanDerm’s performance across various clinical tasks, skin cancer diagnostics, phenotyping, patient risk stratification, inflammatory skin disease diagnostics, lesion segmentation, metastasis prediction, prognosis, and more. The study determined that PanDerm achieved state-of-the-art performance across all tested tasks and outperformed existing models, even when using less than 10% of the labeled data. In real-world clinical settings, PanDerm outperformed dermatopathologists by 10.2% in the identification of early-stage melanoma. Combined with a human-in-the-loop, the model enhanced dermatopathologist performance accuracy by 11% [51].

On 3 April 2025, Paige’s PanCancer Detect received FDA Breakthrough Device designation. However, it is important to still note the model’s limitations. Similar to any of the deep learning models, the performance relies on the quality and diversity of its training data, which may not fully represent global populations or rare histological variants. These tools will still require a human in the loop for safe deployment and use. Additional prospective studies will be necessary to validate real-world utility and address potential biases in dataset representation. Future research should focus on training the model to recognize additional dermatopathological conditions beyond malignancies, such as inflammatory dermatoses and infectious skin diseases. Integrating multi-modal data, such as genomic or radiological information, could further enhance its diagnostic and prognostic potential.

Ultimately, PanDerm AI serves as a compelling example of the potential of deep learning in dermatopathology, providing a glimpse into the next generation of the specialty’s practice with AI: scalable, generalizable, and context-aware.

These four major systems illustrate unique strategies for clinical integration. While each model demonstrates strong performance in narrow contexts, none yet combine all of the elements into a unified, fully validated clinical tool.

### 3.5. Other Notable Models

Beyond the major systems described above, there are also several other AI models that are still in the research and development phase that aim to make progress in specialized dermatopathology applications. The three notable examples below illustrate the continuously expanding scope of AI assistance in pathology.

A model developed by Siarov et al. specifically focuses on a challenging and time intensive task for pathologists, namely distinguishing between nodal metastases and benign intranodal nevi during sentinel lymph node (SLN) evaluation for micrometastatic diseases in order to accurately stage melanoma [46]. This model was trained on only 485 whole slide SLN images, incorporating 5956 pixel wise annotations to maximize specificity and sensitivity. It demonstrated an AUC of 0.965 for nodal metastasis detection. For reference, the range of pathologist performance on this same task ranges from 0.94–0.98. This algorithm also demonstrated a moderate ability to differentiate between nodal metastasis and intra nodal nevi, achieving an AUC of 0.781. A key utility offered by this model is the ability to provide quantitative metrics such as metastatic deposit size, which is vital for cancer staging [46]. This model exemplifies the potential of AI to reduce diagnostic variability and increase throughput in a task central to dermatopathology but frequently limited by workforce capacity and diagnostic ambiguity.

Another critical task in histopathology for assessing tumor burden and margin involvement is tumor segmentation. Opposed to classification models which can output broad diagnostic categories, segmentation models can provide pixel-wise delineations of tumor regions, facilitating morphometric analysis and offering a detailed spatial map of diseases [52,53]. A recent model from Wang et al. uses an encoded-decoder architecture with octave convolutions to simultaneously process high and low frequency image features. Although it was originally developed for liver tumor segmentation, this model now has broader relevance to skin pathology in tasks such as delineating margins or depth in melanoma [52,54]. In terms of performance, the model achieved a mean Dice coefficient of 0.85 and an accuracy of 91%, requiring only 3.2 s per image compared to the minutes or hours needed for the manual process. Similar segmentation models could also serve as preprocessing tools for downstream analytics such as tumor infiltrating lymphocyte quantification or even automated grading systems, and as dermatopathology continues towards increasingly digitized and automated workflows, tools like this will increase the objectivity and reproducibility of analysis.

Another study by Coudray et al. explored whether deep convolutional neural networks (DCNNs) could surface novel histological features predictive of clinical outcomes in osteosarcoma resections [55]. Using a DCNN trained on H&E-stained WSIs, the model was tasked with identifying slide regions associated with poor prognosis. The tiles most predictive of worse outcomes did not always align with canonical histologic patterns; instead, the model highlighted less conventionally appreciated or potentially missed features such as lacy necrotic bone and fat necrosis near the tumor bed. These observations prompted a re-evaluation of the tumor microenvironment and suggested new prognostic biomarkers that may have been overlooked by routine pathologic review. This approach underscores the ability of AI not only to match pathologist-level diagnostic performance, but also to uncover latent morphologic signals with clinical relevance [55].

These three models demonstrate the expanding ability, functionality, and use of AI in dermatopathology, and while not currently integrated into routine clinical practice, the technical sophistication and high performance of these models indicate potential future clinical deployment. Further validation and regulatory processes are likely to not only enhance the diagnostic efficiency but also the standardization and safety of their assessments, and their integration will ultimately support more precise, rapid, objective, and data-driven patient care.

Finally, emerging investigation into computational pathology has explored the use of deep learning to predict immunohistochemical and molecular markers directly from H&E-stained slides. Rubinstein et al. demonstrated that deep learning analysis of colon cancer histopathology accurately detects microsatellite instability (MSI) without molecular testing simply by analyzing tumor morphology [56]. Gerwert et al. combined AI with infrared label-free imaging to classify MSI status in early colon cancers, achieving high sensitivity while bypassing conventional IHC [57]. These studies suggest that morphological features contain latent signals correlated with genetic alterations, which can be captured by advanced deep learning architectures. In parallel, Xing et al. explored whether generative AI could synthetically recreate immunofluorescence (IF) CellPainting images from standard brightfield images [58]. Their findings underscore the potential of generative models to simulate multiplex stains, enabling new possibilities for synthetic histology in dermatopathology, where stains like p53, Ki-67, and Melan-A are commonly used but resource-intensive.

Despite the majority of dermatopathology models being developed are used for neoplastic conditions, recent efforts explore the use of deep learning for inflammatory dermatoses as well. Bao et al. developed a model capable of classifying subtypes of superficial perivascular dermatitis, achieving high accuracy when distinguishing between psoriasiform, spongiotic, and interface patterns [54]. Paige’s PanDerm model has also shown potential advancements towards inflammatory disease phenotyping, though research and development are still ongoing and extensive external validation still remains insufficient.

## 4. Challenges and Limitations of Deep Learning Models in Dermatopathology

Despite the advancements of CNNs, ViTs, and hybrid models in dermatopathology, several key limitations must be addressed to promote their adoption and widespread use in clinical practice.

### 4.1. Dataset Novelty and Bias

The most pressing challenges in all artificial intelligence and deep learning models is dataset limitation. While progress in this field has been facilitated by large annotated datasets with rigorous performance metrics (TCGA, PAD-UFES-20, ISIC), many models continue to be trained on the same images and lack novel annotated sources. For example, Esteva’s dataset and ISIC repositories disproportionately represent lighter skin tones, which may explain performance drops when models are externally validated on darker phenotypes [59]. Even if the architecture and software written by a deep learning researcher is state-of-the-art, the model will only perform as well as its training dataset. Although the number of images available for free–use training is high, they have been frequently reused when training different models. Consequently, many models cannot detect novel clinical presentations or edge cases that may have been identified with a more varied dataset [60]. Dermatopathology AI cannot be reliably generalized without prospectively curated, multi-ethnic datasets.

### 4.2. Explainability and Interpretability

A significant limitation to all deep learning models across medicine, especially dermatopathology, is the black-box nature of how these models process and think about information received [61]. While CNNs and transformers achieve high diagnostic accuracy, their decision-making processes are often opaque, making it difficult for clinicians to understand why a decision and prediction was made. In machine learning, understanding why a decision was made is referred to as explainability. The lack of explainability is a fundamental problem in using deep learning and AI in medicine. Providers must justify conditions through diagnostic conclusions and evidence. Without explainability, it is difficult for providers to use these models in real-world clinical workflows due to liability risks. For example, a saliency map in melanoma histopathology may often highlight adjacent dermis or inflammatory infiltrates rather than atypical melanocytes, and without a clear description of the model’s decision-making, pathologists will lose trust. Techniques such as attention heatmaps and class activation maps (CAM) offer explainability in model behavior, yet there is still significant room for improvement. For deep learning and AI to be effectively employed in real-world use cases and gain widespread trust among dermatopathologists and patients, they must provide interpretable outputs that align with diagnostic criteria and histopathological features used by pathologists in manual interpretation [62].

### 4.3. Government Regulation

Regulatory approval remains a critical hurdle for the clinical adoption of dermatopathology AI. To date, most cleared systems have been classified as assistive tools—technologies that support, but do not replace, human diagnostic judgment. Existing approved products, such as DermAssist and SkinVision, focus primarily on clinical imaging and operate within the regulatory bounds of Clinical Decision Support (CDS) frameworks, where final clinical decisions are left to the clinician [63].

In contrast, an autonomous AI system—intended to render a primary dermatopathology diagnosis without human oversight—would almost certainly be regulated as a Class III device requiring pre-market approval. This will require extensive multi-center clinical trials, external validation, and compliance with Good Machine Learning Practice (GMLP). Researchers and developers must also address the risk of model drift, protected health information security risks, and post-market surveillance requirements [64]. Despite some deep learning models and AI tools receiving clearance through the 510(k) FDA pathway, these models are typically used for narrow, specific disease detection cases and/or research uses. No dermatopathology AI model has yet reached this evidentiary threshold. This distinction between assistive and autonomous diagnosis underscores the progress made and the challenges that remain in translating AI to independent clinical systems.

Dataset novelty and bias, explainability or interpretability, and government regulation are all interrelated: biased datasets worsen interpretability by embedding spurious associations, while regulatory approval requires explainability and robust validation across diverse cohorts. Addressing these barriers will therefore require coordinated strategies that span data curation, algorithm design, and regulatory science.

## 5. Conclusions

Deep learning has evolved from simple CNNs to advanced transformer-based foundation models that demonstrate performance comparable to expert dermatopathologists in specific, well-studied tasks. From lesion classification to pixel-level tumor segmentation, deep learning models have developed to be non-inferior in performance and in some cases outperformed humans, albeit typically in narrow, controlled tasks. Existing deployed models are already enhancing triage efficiency, turnaround times, and serve as a safety net for missed diagnoses, further emphasizing AI’s additive value. Regardless, unanimous adoptions amongst providers remain limited due to three major roadblocks that must be addressed. First, data stewardship: current training corpora overrepresent certain conditions and skin tones, prompting performance gaps for rarer diagnoses and under-represented populations. Prospective, multi-institutional datasets that reflect global diversity in skin types, lesion subtypes, and staining protocols are urgently needed to fill these gaps. Second, the common problem amongst all artificial intelligence models: interpretability and trust. Black-box predictions expose health systems and healthcare providers to legal and malpractice risk. Next-generation models must align model saliency with histopathologic ground truth and human diagnostic heuristics. Advances in computational dermatopathology has shown that deep learning models and artificial intelligence have grown beyond neoplastic classification to include tasks such as sentinel lymph node evaluation, tumor segmentation, analysis of inflammatory dermatoses, and molecular marker prediction; all further emphasizing the need to ensure rigorous verification for more complex tasks. Lastly, regulatory and post-market oversight must be clarified and complied with. For eventual autonomous deployment, models must demonstrate durable and longitudinal performance at the highest standards in a way that prevents model drift. Pivotal, prospective multi-site clinical trials will be required to meet FDA evidentiary thresholds. Addressing these hurdles will ultimately set up deep learning in dermatopathology for success and motivate all stakeholders involved in healthcare to promote the adoption of AI within dermatopathological care.

## Figures and Tables

**Table 1:** Performance and Architectures of Deep Learning Models Trained on Dermoscopy for Dermatopathology.

Model Name	Architecture Type	Dataset(s) Type Used	AUROC	Accuracy	Sensitivity	Specificity
**Esteva et al. CNN** [3]	CNN	Dermoscopy (Various Datasets)	0.94	72.1%	—	—
**ResNet-152 (Behara et al.)** [26]	Hybrid CNN-Attention	Dermoscopy (HAM10000 and ISIC)	—	93.1%	94.9%	92.8%
**CNN + Random Forest (Ba et al.)** [14]	Hybrid CNN-Random Forest	Dermoscopy	0.998	98.2%	100%	96.5%
**ConvNeXtV2-Transformer (Ozdemir & Pacal)** [23]	Hybrid ConvNeXtV2-Transformer	Dermoscopy (ISIC 2019)	—	93.6%	90.7%	93%

**Table 2:** Performance and Architectures of Deep Learning Models Trained on Histopathology for Dermatopathology.

Model Name	Architecture Type	Dataset(s) Type Used	AUROC	Accuracy	Sensitivity	Specificity
**Hekler et al. CNN** [5]	CNN	Histopathology (Melanoma vs. Nevus Slides)	—	68%	76%	60%
**ViT for SCC Margin Assessment (Park et al.)** [20]	ViT	Histopathology (Squamous cell carcinoma margins)	0.927	92.8%	89%	91%

**Table 3:** Performance and Architectures of Deep Learning Models Trained on Whole Slide Images for Dermatopathology.

Model Name	Architecture Type	Dataset(s) Type Used	AUROC	Accuracy	Sensitivity	Specificity
**Xie et al. CNN** [12]	CNN	Whole Slide Images	0.986	93%	93.8%	95.7%
**Graph-Transformer (GTP, Zheng et al.)** [10]	Hybrid CNN-Transformer	Whole Slide Images (TCGA (Lung), 4818 WSIs)	0.965	93.5%	91.9%	96%

**Table 4:** Summary of PanDerm AI: Design, Capabilities, and Deployment Considerations.

Component	Description
**Model Name**	PanDerm AI [48]
**Type**	Multimodal Foundation Model for Dermatology
**Training Data**	>3 million images from 11 institutions across 4 imaging modalities
**Modalities**	Clinical images; Dermoscopy; Histopathology; Smartphone photos
**Tasks Evaluated**	28 total [51]: Skin cancer detection; Inflammatory conditions; Lesion segmentation; Change monitoring; Prognosis prediction
**Evaluation Benchmarks**	Outperformed prior models across all 28 tasks
**Real-World Validation**	Improved early-stage melanoma detection by 10.2% over clinicians
**Human-AI Collaboration**	Improved multiclass skin cancer diagnostic accuracy by 11% with clinician input
**Key Limitations**	Requires diverse training data; Needs human-in-the-loop; Prospective validation needed
**Future Directions**	Expand to inflammatory, infectious, autoimmune dermatoses; Integrate multimodal data (e.g., genomics, clinical notes)

## Data Availability

No new data were created or analyzed in this study.
